# Preparation and Evaluation of Antimicrobial Properties and Cytotoxic Potentials of Nutmeg and Tulsi Gel

**DOI:** 10.7759/cureus.44140

**Published:** 2023-08-25

**Authors:** Vyshnavi B Sindhusha, Sankari Malaiappan, Rajesh S Kumar

**Affiliations:** 1 Periodontology, Saveetha Institute of Medical and Technical Sciences, Chennai, IND

**Keywords:** local drug delivery, cytotoxic potential, antimicrobial activity, tulsi, nutmeg, periodontitis

## Abstract

Aim: The aim of the study is to prepare the dual gel using nutmeg and Tulsi and then the evaluation of the antimicrobial properties and cytotoxic potential.

Materials and methods: The Nutmeg Tulsi gel preparation has been done with a mixture of equal amounts of nutmeg and Tulsi powder. To the above-mentioned mixture, 5 mL of the concentrate is added and mixed thoroughly until the gel formation is done. The antimicrobial property is checked in the *Porphyromonas gingivalis* organism (p>0.05). The cytotoxic potential is checked in the Brine variety of the shrimp. The statistical analysis is done using a Paired t-test.

Results: The results stated that the Nutmeg Tulsi gel at a concentration of 100 microgram/mL showed a greater zone of inhibition (4.1±0.09 mm) when compared with doxycycline and has high antimicrobial potential in both *Aggregatibacter actinomycetemcomitans *and *Porphyromonas gingivalis.*

Conclusion: The antimicrobial property of Nutmeg Tulsi gel has been demonstrated to be effective against *P. gingivalis* and *A.* *actinomycetemcomitans*. This suggests that it could be used as an affordable and effective "adjunct" alongside standard care for managing periodontal conditions.

## Introduction

Living organisms including human beings are being consistently subjected to potentially harmful pathogens throughout their lives. This persistent exposure encompasses a vast array of diseases that profoundly impact human health. Despite possessing evolved defense mechanisms to combat these challenges the initial line of defense often proves inadequate. Consequently, relying on chemotherapeutic agents becomes necessary in tackling pathogen-induced infections. Historical precedent demonstrates the discovery or synthesis of numerous chemical agents designed for treating and curing such infections. Now it’s imperative that we explore alternative avenues in lieu of synthetic antibiotics and adjunctive drugs [1].

Spices and aromatic herbs offer a promising solution to the aforementioned problems due to their recognized antioxidant and antimicrobial qualities [2]. Furthermore, these natural remedies have long played an essential role in traditional medicine across various cultures. An example of such an herbal resource is nutmeg - obtained by drying the kernel of an ovoid seed called Myristica fragrans. It holds multiple uses; it serves as a popular spice while also being employed in alternative medicine for its reputed aphrodisiac effects. Memory-enhancing properties, ability to alleviate diarrhea, anti-inflammatory attributes, and even potential in fighting cancer. Osmium sanctum is indeed a highly revered plant in traditional medicine, particularly in India.

Tulsi has a history of use in traditional medicine, the plant has been known for its medicinal values. The leaves, in particular, are commonly used in different forms. Aqueous extracts can be obtained from fresh or dried leaves, which are then used in herbal teas. The medicinal properties attributed to Tulsi are diverse. Tulsi possesses various properties such as antimicrobial, anti-inflammatory, and antioxidant. Tulsi in the form of essential oils is mainly known for its antimicrobial activity [3]. Tulsi is considered beneficial for various ailments, including respiratory disorders, digestive issues, skin conditions, and cardiovascular problems. It is also used for its potential anti-cancer properties and for promoting longevity.

Tulsi has been traditionally used to treat different systemic diseases. The extracts of Tulsi have been used in the treatment of various conditions, including poisoning, stomachache, and common colds as it is often used to alleviate symptoms of the common cold, such as cough, congestion, and sore throat. Its antimicrobial and expectorant properties may help in providing relief to headaches, malaria, inflammation, and heart disease. Its hypotensive, hypolipidemic, and antioxidant properties may contribute to its potential cardiovascular benefits. In addition to the aqueous extracts, oils derived from Tulsi are also believed to possess various useful properties, such as expectorant, analgesic, antiemetic, antipyretic, anti-asthmatic, hypoglycemic, hepatoprotective, and immune modulators. Tulsi has been used in various formulations such as essential oils and can be used against various microorganisms [4-6]. Tulsi is also known for its property of reducing elevated carbon dioxide (CO_2_) levels [7].

The essential oil of Tulsi is the most commonly used to check the antimicrobial properties [8]. The combination of nutmeg and Tulsi in traditional medicine is additive, as both herbs have been recognized for their potential health benefits. Nutmeg (Myristica fragrans) is a spice commonly used in culinary applications, and Tulsi has a long history of traditional medicinal use, as we discussed earlier. Tulsi is known to possess antibacterial compounds that are effective against various anaerobes such as pseudomonas [9].

However, the additive potential of both nutmeg and Tulsi in terms of their antioxidant and antimicrobial properties has not been specifically assessed. The different phytochemicals of both herbs are known for their diverse biological activities and may contribute to the observed health benefits of nutmeg and Tulsi. Specifically, the compounds found in these herbs can act as antioxidants through mechanisms such as metal chelation and inhibition of lipid peroxidation. These antioxidants help protect the body against oxidative stress, which is associated with various chronic diseases. Furthermore, both nutmeg and Tulsi have antimicrobial properties, which may be attributed to their bioactive compounds, and are effective against various gram-positive organisms such as *Staphylococcus aureus* [10]. Antimicrobial activity can help inhibit the growth of microorganisms, including bacteria, fungi, and viruses. Doxycycline inhibits bacterial protein synthesis by allosterically binding to the 30S prokaryotic ribosomal subunit, thus possessing the bacteriostatic property. The aim of the current study is to prepare the dual gel using nutmeg and Tulsi and then the evaluation of antimicrobial properties and cytotoxic potential.

## Materials and methods

Preparation of the gel

**Figure 1:**
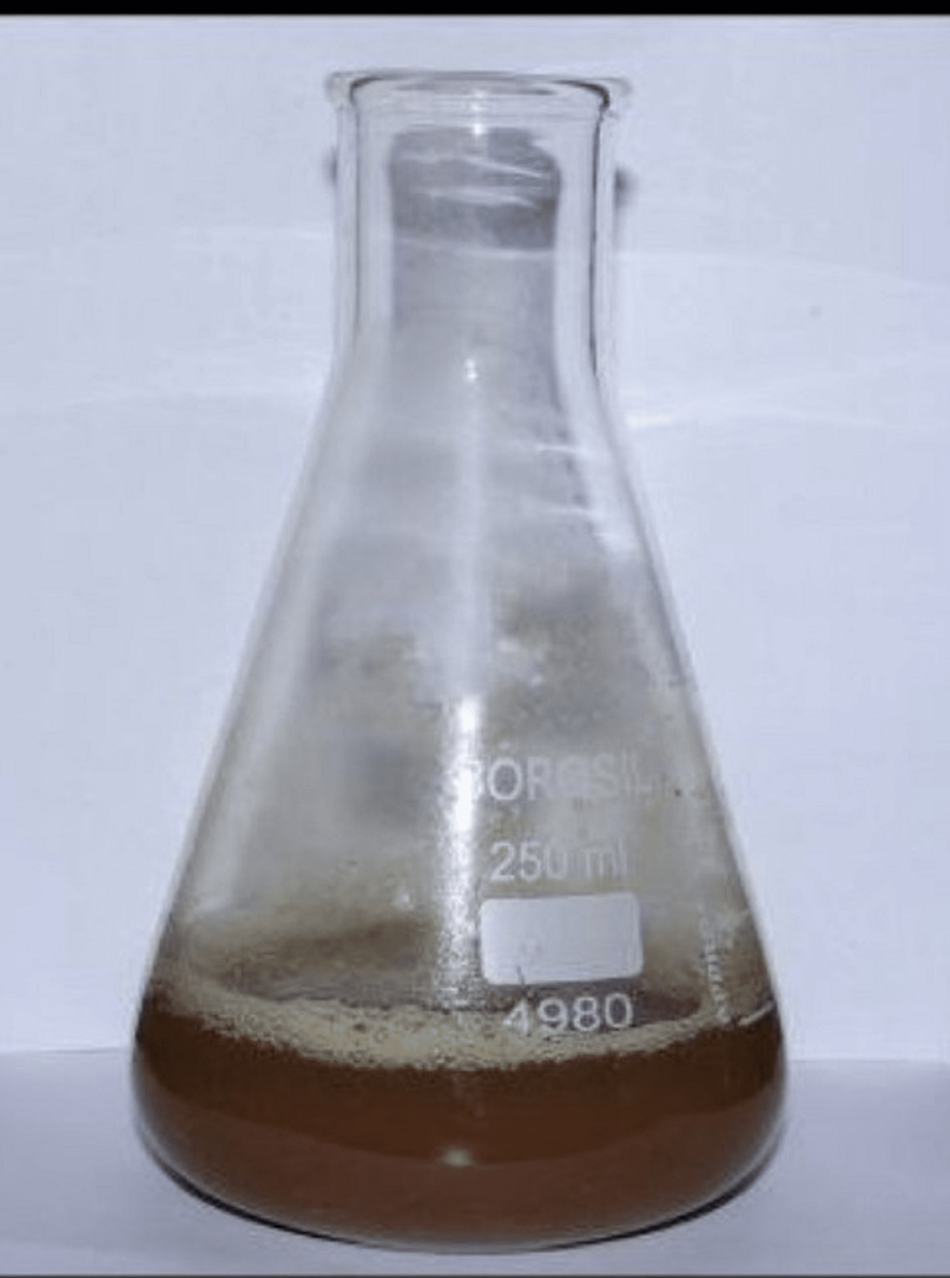
Nutmeg Tulsi extract The image describes the crude extract of nutmeg and Tulsi gel.

The nutmeg and Tulsi gel has been prepared by taking an equal quantity of plant extracts obtained from commercial products available of nutmeg and Tulsi in the market. The institutional clearance for this research has been given by the Department of Nanotechnology, Saveetha Dental College, and the 5 grams of nutmeg powder is mixed with 5 grams of Tulsi powder which 100 mL of distilled water has been mixed and the obtained solution has been heated at 65 degrees centigrade for 15 minutes to obtain a thick crude extract of Nutmeg Tulsi (Figure 1).

**Figure 2:**
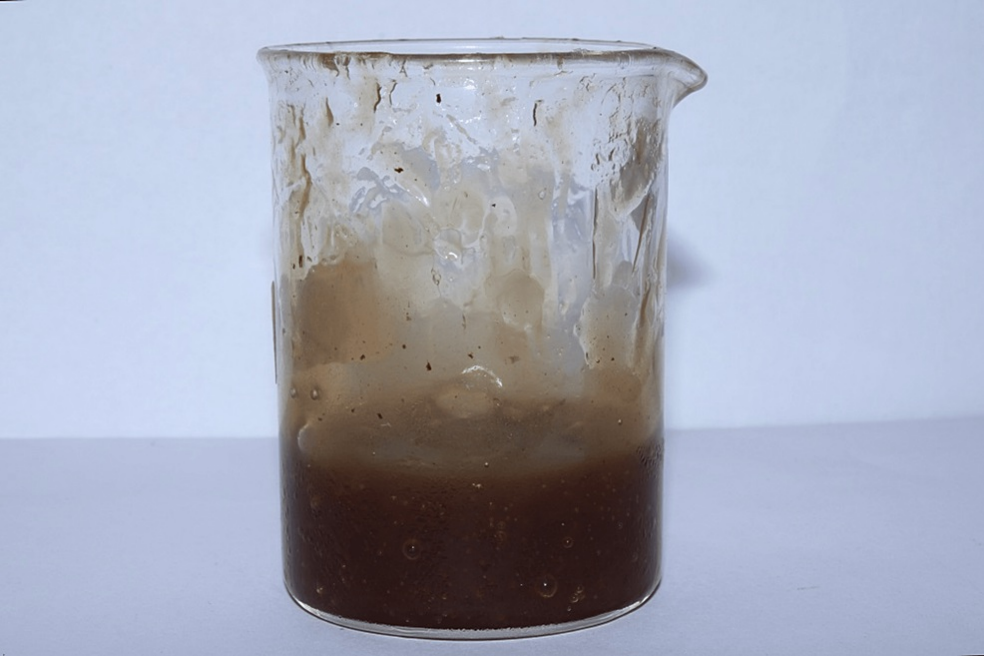
Nutmeg Tulsi gel The 5 mL Nutmeg Tulsi concentrate has been mixed with the Carbopol and carboxymethylcellulose extract to obtain the Nutmeg Tulsi gel.

Then 1.5 grams of carboxymethyl cellulose and 1.5 grams of Carbopol are prepared into a thick mixture using 20 mL of distilled water. To this Carbopol and carboxymethyl cellulose mixture, 5 mL of Nutmeg Tulsi concentrated extract has been added to obtain Nutmeg Tulsi gel (Figure 2).

Antimicrobial activity

One gram of nutmeg and Tulsi gel has been dissolved in 10 mL of distilled water to obtain a dilution of nutmeg and Tulsi gel in micrograms. A total of four samples have been taken with concentrations of 25 micrograms, 50 micrograms, and 100 micrograms of Nutmeg Tulsi gel are also made. These different gel dilutions have been collected and inoculated onto the agar plates for microbial assays. The Nutmeg Tulsi gel has been used as an experimental group and doxycycline has been used as a control group. The culture of the plates was done in a germ-free environment to check their antimicrobial property against gram-positive bacteria.

Cytotoxic potential

**Figure 3:**
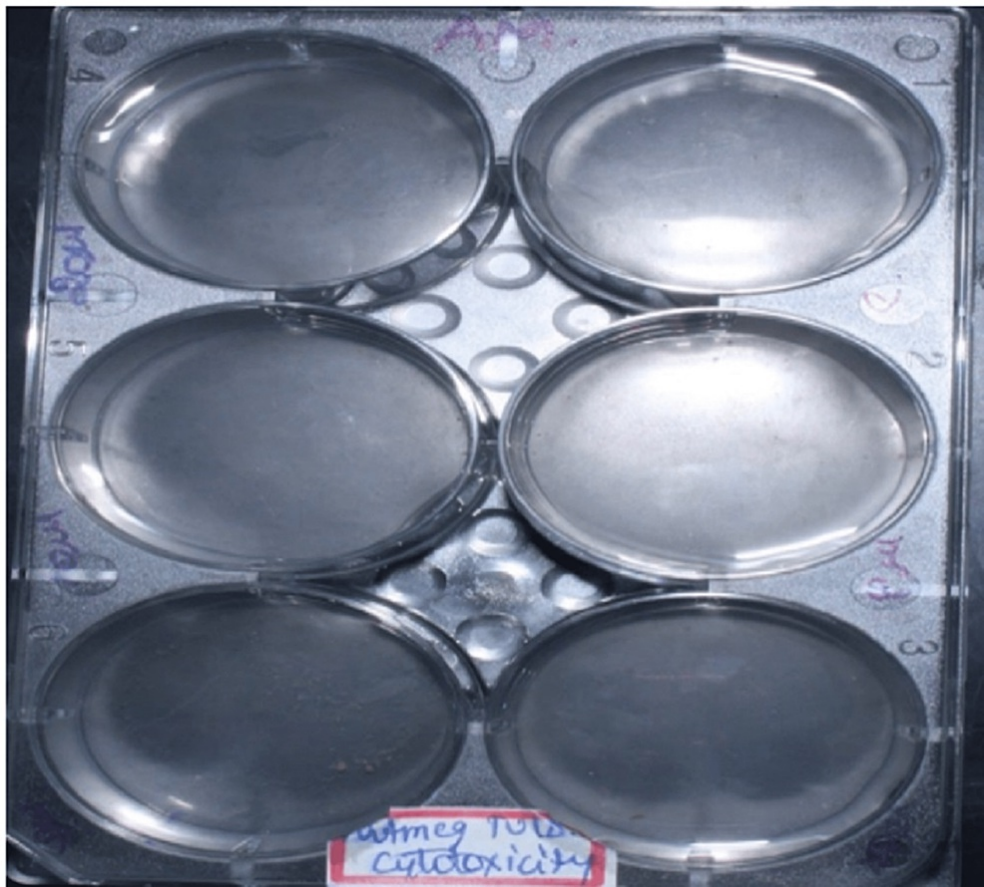
The cytotoxic potential of the Nutmeg Tulsi gel checked in the Brine variety of shrimp.

The cytotoxic potential of the nutmeg and Tulsi gel has been checked in the Brine variety of the shrimp (IHEC/SDC/PERIO-2104/23/100). The Brine shrimp were seeded on a six-well plate with 10 shrimp per well in 100 microliters of the growth medium, then the shrimp were incubated for a time period of 24 hours at 37 degrees centigrade. The nutmeg and Tulsi gel was added in different concentrations such as 10 microliter, 20 microliter, 40 microliter, and 80 microliters by diluting gel with distilled water along with the control group and shrimp were incubated for 12 hours at 37 degrees (Figure 3).

## Results

Antimicrobial activity

**Figure 4:**
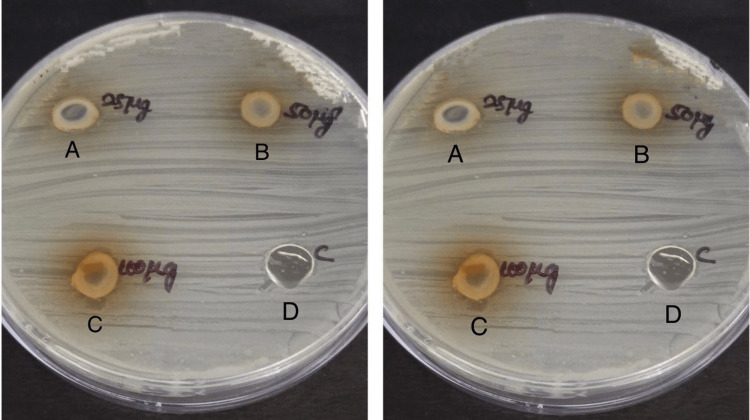
Antimicrobial activity against A. actinomycetemcomitans The antimicrobial activity is seen against *A. actinomycetemcomitans* in agar medium with A having 25 microgram concentration of Nutmeg Tulsi gel, B having 50 microgram concentration of Nutmeg Tulsi gel, C having 100 microgram concentration of nutmeg and Tulsi gel, and D is the control (doxycycline).

**Figure 5:**
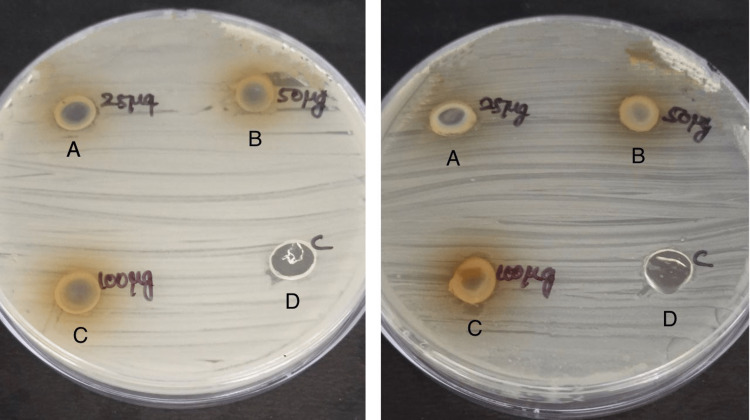
Antimicrobial activity against P. gingivalis The antimicrobial activity is seen against *P. gingivalis* in the agar medium with A having 25 microgram concentration of Nutmeg Tulsi gel, B having 50 microgram concentration of Nutmeg Tulsi gel, C having 100 microgram concentration of nutmeg and Tulsi gel, and D is control (doxycycline) concentrations of Nutmeg Tulsi gel.

The microbiological test is performed to evaluate the antimicrobial effect of Nutmeg Tulsi gel against specific bacteria: *A. actinomycetemcomitans* and *P. gingivalis* which are isolated from the plaque sample collected in periodontitis patients. The agar well diffusion method was used as this method helps in the assessment of the zone of inhibition of Nutmeg Tulsi gel against doxycycline, which is a commonly used antibiotic as it inhibits bacterial protein synthesis by allosterically binding to the 30S prokaryotic ribosomal subunit (Figures 4A-4C, 5A-5C).

The mean in the widths of the zone of inhibition for different concentrations of Nutmeg Tulsi gel and doxycycline in agar plates inoculated with *A. actinomycetemcomitans* and *P. gingivalis* were listed in Table 1.

**Table 1:** Mean values and standard deviations (SD) for the width of zones of inhibition in mm The table indicates the mean value of the zone of inhibition along with the standard deviation for different concentrations of Nutmeg Tulsi gel along with doxycycline (control).

Different Concentrations of Test Material	Mean Width of Zone of Inhibition (mm)	Standard Deviation (mm)
25 micrograms (Nutmeg Tulsi gel)	1.8	0.87
50 micrograms (Nutmeg Tulsi gel)	12.5	0.40
100 micrograms (Nutmeg Tulsi gel)	4.1	0.09
Doxycycline (CONTROL)	1.2	0.07

The mean width of the zone of inhibition obtained around different microorganisms such as *A. actinomycetemcomitans* and *P. gingivalis* with different concentrations of Nutmeg Tulsi gel against doxycycline were compared using the Paired t-test, and the results are shown in Table 2.

**Table 2:** Comparison of mean width of zone of inhibition using paired t-test This table gives the comparison of mean width of zone of inhibition along with the asymptomatic significance.

Organism	25 micrograms	50 micrograms	100 micrograms	Control
P. gingivalis				
Z	-1.842	-2.612	-3.422	-1.734
p Value	*0.006	*0.017	*0.015	0.003
(2 tailed)				
A. actinomycetemcomitans				
Z	-1.384	-2.446	-4.382	-1.832
p Value	*0.019	*0.016	*0.005	*0.063
(2 tailed)				

The results of the study indicated that at concentrations of 50 micrograms and 100 micrograms, Nutmeg Tulsi gel has shown greater antimicrobial activity against *A. actinomycetemcomitans* and *P. gingivalis* when compared with doxycycline. The zone of inhibition produced by Nutmeg Tulsi gel extract at these concentrations was greater when compared with those produced by doxycycline, and the difference between them was statistically significant (p>0.05).

Cytotoxic potential

The effect of the nutmeg and Tulsi gel on the brine shrimp was determined by using a presto-blue test. The brine shrimp were treated with various dilutions of the nutmeg and Tulsi gel from 5 microliter to 80 microliter, and the results state the shrimp viability (cytotoxic potential) against the dilution of the Nutmeg Tulsi gel. More than 50% of the shrimp are viable even when the concentration is 80 microliters. When the nutmeg and Tulsi concentrate in the gel are at 80 mg/mL, the shrimp viability is more than 50% and therefore Nutmeg Tulsi gel is not considered cytotoxic.

## Discussion

The effect of nutmeg and Tulsi extract on cell viability was assessed using the Presto-Blue test, a commonly used method to evaluate cell metabolic activity and viability. The cells were exposed to different concentrations of the extract, ranging from 10 to 80 mg/mL. The results of the study stated that the cytotoxicity of the extracts decreased with increasing concentration. In other words, as the concentration of the extract increases, the negative impact on cell viability is not affected by more than 50%. This suggests that the extracts of nutmeg and Tulsi were less harmful to the cells at their higher concentrations. Specifically, the study found that even at the highest tested concentration of 80 mg/mL, the cell viability decreased by less than 50%. This means that the cells retain at least 50% of their metabolic activity and viability when treated with this concentration of the extract [11]. As a result, the concentration of 80 mg/mL was considered non-cytotoxic in this context. When the aqueous extract of Tulsi was used at a concentration of 80 mg/mL, was found to be less cytotoxic. It suggests that these concentrations did not significantly decrease cell viability, as the decrease observed was less than 50%.

In the present study, it has been observed that there is a significant antimicrobial activity of nutmeg extracts against the tested microorganisms. The acetone extract of nutmeg has exhibited the highest antimicrobial activity when compared with other extracts of nutmeg. This finding states that the nutmeg seeds possess potent antimicrobial properties against bacteria and fungi [12].

Nutmeg contains various bioactive molecules that have been isolated and found to have antimicrobial importance. These compounds likely contribute to the observed antimicrobial activity. Further research is needed to identify and characterize the specific antimicrobial compounds present in nutmeg seeds. These antimicrobial compounds mainly play a role in the antimicrobial activity of both nutmeg and Tulsi gel [13]. Understanding the chemical composition and mechanisms of action of these compounds can provide valuable insights into their potential applications in antimicrobial therapies.

The additional information regarding the antimicrobial compounds found in nutmeg includes the key findings mentioned Trimyristin and myristic acid: Narasimhan and Dhake [13] reported that compounds such as Trimyristin and myristic acid are the chief antibacterials extracted from nutmeg. These elements are believed to play a major role in the antimicrobial activity of nutmeg against bacteria. Three lignans were isolated from the methanolic extract of nutmeg. These lignans were found with antifungal properties, indicating their potential role in combating fungal infections. Takikawa has found the antimicrobial activity of nutmeg against *Escherichia coli* O157 strain [14]. They found that the *E. coli* O157 strain was highly sensitive to β-pinene, a compound present in nutmeg. This suggests that β-pinene contributes to the antimicrobial effects against this specific strain of bacteria. The compounds present in nutmeg may also contribute to the overall antimicrobial properties of nutmeg. These findings highlight the presence of various bioactive compounds in nutmeg, such as trimyristin, myristic acid, lignans, b-pinene, and plant phenolics, which likely play a role in its antimicrobial activity against bacteria and fungi.

The α and β variants of pinene-type hydrocarbons present in nutmeg are reported to possess antimicrobial activity. They are believed to contribute to the antimicrobial effects by potentially disrupting cell membranes through their lipophilic properties. Carvacrol is an important component of nutmeg and has been reported to exhibit antimicrobial activities. It is known to have the ability to diffuse through cell membranes and enter inside the cell, where its interaction with intracellular sites is critical for its antimicrobial effects. It has been reported that p-cymene works synergistically with carvacrol. Together, they have the ability to expand the bacterial membrane, leading to membrane destabilization and ultimately causing antimicrobial effects. The combination of p-cymene and carvacrol, as well as the presence of b-caryophyllene, contributes to the overall antimicrobial and health-promoting properties of nutmeg.

The agar diffusion method and the dilution method are two commonly used techniques for examining the antimicrobial activity of herbs such as Tulsi. The agar diffusion method involves creating wells soaked with the Tulsi onto an agar plate inoculated with microorganisms. Antimicrobial activity is determined by measuring the zone of inhibition, which is the area around the well or disk where the growth of microorganisms is inhibited [15]. The evaporation of the volatile compounds present in the herbs during the experimental period affects the accuracy and reliability of the results. To overcome this issue, the dilution method is often preferred for assessment of the antimicrobial activity of herbs. In the dilution method, the herbal compounds are diluted in a liquid broth or incorporated into agar at various concentrations. The microorganisms are then exposed to these dilutions, and the minimum inhibitory concentration (MIC) is detected, which is the lowest concentration of the herbal compounds that inhibits the growth of microorganisms. Kalemba and Kunicka (2003) have done a study that stated that the MIC and minimum bactericidal concentration (MBC) of the herbal compounds are well determined in broth dilution methods with serial dilutions of the herbal compound [16].

Regarding the bacteriostatic activity of nutmeg and Tulsi gel at levels of 100 μg/mL highest antibacterial activity is seen against gram-negative bacteria such as *P. gingivalis* and *A. actinomycetemcomitans*. The antimicrobial activity of Nutmeg Tulsi gel is attributed to variations in the bacterial strains used, the concentrations and formulations of the Nutmeg Tulsi gel, as well as the specific antimicrobial mechanisms involved [17]. Further research is necessary to elucidate the underlying mechanisms of action and to evaluate the efficacy of Nutmeg Tulsi gel against a broader range of bacterial strains. The study done by Mishra and Mishra (2011) stated a better inhibition of gram-positive organisms using optical density reduction as the measure of activity. The *S. aureus* species exhibited slightly better activity under the test conditions [18]. The difference in the antimicrobial activity of Tulsi compounds is due to variations in the composition of volatile compounds present in the Tulsi as the Tulsi is cultivated from different geographical places. Overall, the observed variations in the antimicrobial activity of Nutmeg Tulsi gel are influenced by multiple factors such as the composition of volatile compounds, geographical source, specific cultivars, and the inherent resistance of certain bacterial strains.

Limitations of the study

The above-mentioned study is an in vitro study where only two properties - antimicrobial activity and cytotoxic potential - have been checked. Even though the nutmeg and Tulsi gel has given favorable results, further studies need to be conducted to check other properties of the gel.

## Conclusions

The synergistic combination of nutmeg and Tulsi gel has been shown to possess potent antimicrobial activity against common periodontal pathogens such as *P. gingivalis *and *A. actinomycetemcomitans* and it has also shown less cytotoxicity even at higher concentrations. Thus, this novel gel evaluated in the present study may help in the prevention and treatment of microbial infections affecting the periodontium, such as gingivitis and periodontitis. However, further investigations are needed for its application in vivo.
